# Analysis of Microbial Community Structure and Diversity in Burial Soil of Yangguanzhai Cemetery

**DOI:** 10.3389/fmicb.2022.845870

**Published:** 2022-05-31

**Authors:** Xiaoyang Wei, Xue Ling, Liping Yang, Jun Zhang, Menghe Cui, Zhang He, Xichen Zhao, Manli Sun

**Affiliations:** ^1^School of Cultural Heritage, Northwest University, Xi’an, China; ^2^Shaanxi Academy of Archaeology, Xi’an, China; ^3^School of Life Sciences, Northwest University, Xi’an, China

**Keywords:** Yangguanzhai Cemetery, human bone, high-throughput sequencing, microorganism, community

## Abstract

As one of the common physical remains in archaeological discoveries, human bones are important bases for studying the history of human development, which is of great significance for exploring the evolution law of ancient human, reconstructing ancient human society, and tracking the development of human civilization. However, in the process of human bone burial, in addition to being affected by physical and chemical factors, it will also be affected by microorganisms in the buried soil, resulting in a variety of diseases. According to the determination and analysis of the microbial community structure and diversity in the burial soil of Yangguanzhai Site in Gaoling District in Xi’an city, Shaanxi Province, this paper attempts to explore the influence of microorganisms in the burial environment on human bones, in order to provide scientific proof for the microbial prevention and control of bone relics in the archaeological excavation site. For the first time, Illumina NovaSeq high-throughput sequencing technology was used to analyze the microbial community structure in the burial soil. At the phylum level, there were 8 dominant bacteria species in the soil samples of tombs, which were Firmicutes, Actinobacteriota, Actinobacteria, Proteobacteria, Acidobacteriota, Methylomirabilota, Chloroflexi, Bacteroidota. At the genus level, there were 12 dominant species in the soil samples of tombs, including MIZ17, MND1, Gaiella, oc32, Kroppenstedtia, Halomonas, Bacteroides, Dongia, Faecalibacterium, Nocardioides, Pseudomonas, Pseudonocardia. The overall microorganisms in the soil of Yangguanzhai Cemetery were relatively well-distributed, and the microbial community structure near human bones is the most abundant and diverse. Therefore, it is necessary to take some measures to control microorganisms and protect human bones.

## Introduction

For a long time, the corrosion mechanism and protection methods of cultural relics have been widely concerned by natural science and cultural relics protection academia. Besides physical and chemical factors, microorganisms also play an important role in the corrosion of cultural relics. Scholars have done a lot of research work on the microbial corrosion of cultural relics, such as painted murals (Wu et al., 2013a,b,c; Duan et al., 2019), stone cultural relics (Zhang et al., 2001; Fu et al., 2009; Yan et al., 2012), earthen ruins (Xia et al., 2013; Wu et al., 2014; Wang et al., 2018), textile artifacts (Yan, 2004), paper artifacts (Yan et al., 2011; Tang et al., 2015; Quan, 2019), wooden artifacts (Liu, 2014; Xiao et al., 2014), etc., but there is little research on the impact of microorganisms on bone cultural relics.

As one of the common remains in archaeological discoveries, human bone is an important material basis for studying the history of human development. In recent years, with the progress and development of technology, the research methods of bones are gradually diversified, including bone morphology analysis, bone microstructure analysis, bone pathology analysis, bone chemistry analysis, DNA analysis, etc., which can fully reveal the age, gender, growth and development status, disease, behavior, diet, social relations and other aspects of ancient human information. Therefore, human bones found in archaeological sites play an important role in academic research such as anthropology, history, archaeology, and ethnology, and are of great significance for exploring the evolutionary laws of ancient humans, reconstructing ancient human society, tracking the development of human civilization. It is known that human bones are composed of 70% inorganic substances (mainly calcium phosphate) and 30% organic substances (mainly proteins). The texture structure and protein composition of human bones are easy to absorb moisture, which provide a hotbed for the attachment and breeding of microorganisms. Acid substances and enzymes produced by the growth and metabolism of microorganisms will corrode and decompose inorganic substances in human bones, which not only affects the appearance of human bones, but also may trigger a series of physical and chemical reactions, so that calcium phosphate in bones can be replaced into the soil environment, weakening the mechanical properties of human bones, such as cracking resistance, compression resistance and bending resistance, which is not conducive to long-term stable preservation. Therefore, it is urgent to strengthen the research and protection of unearthed human bones.

Yangguanzhai Site, located in Gaoling District in Xi’an City, Shaanxi Province, is a large central settlement site in the middle and late Yangshao period in Guanzhong area. Yangguanzhai Cemetery is located in the Northeast outside the surrounding trenches of the site, with a total area of more than 90,000 square meters, dating from 5,600 to 4,900 years ago. It is the first adult cemetery of Miaodigou culture discovered and confirmed in China, filling the gaps in archaeological discoveries in related fields. It provides scientific materials for the settlement form, burial custom, ethnic type, blood relationship, social organization status and other major issues of Miaodigou culture (Shaanxi Academy of Archaeology, 2009, 2011, [Bibr B15][Bibr B16]). The important academic value of the Site and Cemetery have also been widely recognized by the academia. In 2008 and 2017, the Site and Cemetery were separately selected as the “top ten new archaeological discoveries in China.”

The human bones unearthed from the Yangguanzhai Cemetery are currently preserved *in situ*, and there are diseases such as soil rust, cracking, calcification, and crisp powder. Traditional microscope observation and study of the diversity and community structure of microorganisms are very limited, and the number of microorganisms that can be obtained by artificial cultivation is even rarer (Solden et al., 2016). High throughput sequencing overcomes the limitations of traditional methods by sequencing the microbial community, comparing with the existing sequences in the database, and analyzing the microbial diversity. The detection results are informative, and some micro strains can be detected (Ronaghi et al., 1998; Goodwin et al., 2016).

Therefore, taking Yangguanzhai Cemetery as an example, we use Illumina NovaSeq high-throughput Sequencing Technology to analyze the community structure and diversity of the bacteria in the tomb fill, trying to explore the impact of microorganisms on human bones in the burial and preservation environment, so as to provide scientific basis for the microbial prevention and control of bone relics in the archaeological excavation site.

## Materials and Methods

### Sample Collection

These samples were collected from Yangguanzhai Cemetery, using the principle of pre sampling. Before the excavation of cultural relics, a reasonable scheme is designed according to the information of the previous investigation of cultural relics, so as to avoid the pollution of the field work and lead to the existence of experimental errors. In the process of archaeological excavation of the target tomb, we adopted the method of sampling while excavating in accordance with the experimental requirements. After the tomb fill was exposed, we took soil samples with sterile spoon, sealed them with sterile Eppendorf tube and stored them in liquid nitrogen tank. After that, they were stored in dry ice and sent to Novogene for subsequent testing. The materials used were all sterilized in advance at the time of sampling. Among them, M277 has been excavated at the time of sampling, and M501 has been excavated and exposed to the air for 1 year.

The sampling information is as follows: (Table 1).

**TABLE 1:** Samples information of tombs in Yangguanzhai Cemetery.

Group	Name	Sampling location
M277.1	M277.1.a	Soil 20 cm from the burials opening
	M277.1.b	Soil 20 cm from the burials opening
	M277.1.c	Soil 20 cm from the burials opening
M277.2	M277.2.a	Soil 30 cm from the burials opening
	M277.2.b	Soil 30 cm from the burials opening
	M277.2.c	Soil 30 cm from the burials opening
M277.D	M277.D.a	Human bone right soil (foot side)
	M277.D.b	Human bone right soil (Pelvic side)
	M277.D.c	Human bone right soil (Cephalic side)
M501.D	M501.D.a	Human bone right soil (foot side)
	M501.D.b	Human bone right soil (Pelvic side)
	M501.D.c	Human bone right soil (Cephalic side)
	M501.G	Small skull fragments
S	s.1	Soil outside burials

### Microbial Species Determination

#### High-Throughput Sequencing of Samples

##### Extraction of Genome DNA

Total genome DNA from samples was extracted using CTAB method. DNA concentration and purity was monitored on 1% agarose gels. According to the concentration, DNA was diluted to 1 ng/μL using sterile water.

##### Amplicon Generation

The primers were designed according to the conserved region. The bacteria were mainly based on 16S region. The V4 variable region of 16S rDNA was amplified using the universal bacterial primer 515F/806R (F5′-AYTGGGGYDTAAAGNG-3′, R5′-AYTGGGYDTAAAGNG-3′). All PCR reactions were carried out with 15 μL of Phusion^®^ High-Fidelity PCR Master Mix (New England Biolabs); 2 μM of forward and reverse primers, and about 10 ng template DNA. Thermal cycling consisted of initial denaturation at 98°C for 1 min, followed by 30 cycles of denaturation at 98°C for 10 s, annealing at 50°C for 30 s, and elongation at 72°C for 30 s. Finally 72°C for 5 min.

##### PCR Products Quantification and Qualification

Mix same volume of 1× loading buffer (contained SYB green) with PCR products and operate electrophoresis on 2% agarose gel for detection. PCR products was mixed in equidensity ratios. Then, mixture PCR products was purified with Qiagen Gel Extraction Kit (Qiagen, Germany).

##### Library Preparation and Sequencing

Sequencing libraries were generated using TruSeq^®^ DNA PCR-Free Sample Preparation Kit (Illumina, United States) following manufacturer’s recommendations and index codes were added. The library quality was assessed on the Qubit^@^ 2.0 Fluorometer (Thermo Scientific) and Agilent Bioanalyzer 2100 system. At last, the library was sequenced on an Illumina NovaSeq platform and 250 bp paired-end reads were generated.

#### Data Analysis

Referring to the Tags quality control process of QIIME (V1.9.1), each sample data is separated from the offline data according to the barcode sequence and PCR amplification primer sequence. After the barcode and primer sequence are intercepted, the reads of each sample are spliced with FLASH (V1.2.7), filtered and processed to obtain high-quality tags data (clean tags). The tags obtained after the above processing need to be processed to remove the chimera sequence, and the tags sequences are compared through the species annotation database to detect chimeric sequences, and finally remove the chimeric sequences to obtain the final effective tags.

The effective sequences were clustered by OTUs (operational taxonomic units) using uparse software (V7.0.1001) at 97% similarity level, and the species annotation analysis was conducted based on the Mothur method and Silva Database (the threshold was set at 0.8–1). The phylogenetic relationships of all OUT (Operational Taxonomic Units) representative sequences were obtained by using music software. Finally, the data of each sample were homogenized for αdiversity analysis. The αdiversity index, UniFrac distance and UPGMA sample cluster tree were calculated by using QIIME (V1.9.1). R software was used to draw the dilution curve, species accumulation curve and αdiversity index analysis. LEfSe software is used for LEfSe analysis, and the filter value of LDA score is 4 by default. The heat map was drawn based on the weighted distance using QIIME (V1.9.1).

## Data Analysis

### Sequencing Data Quality Assessment

**FIGURE 1:**
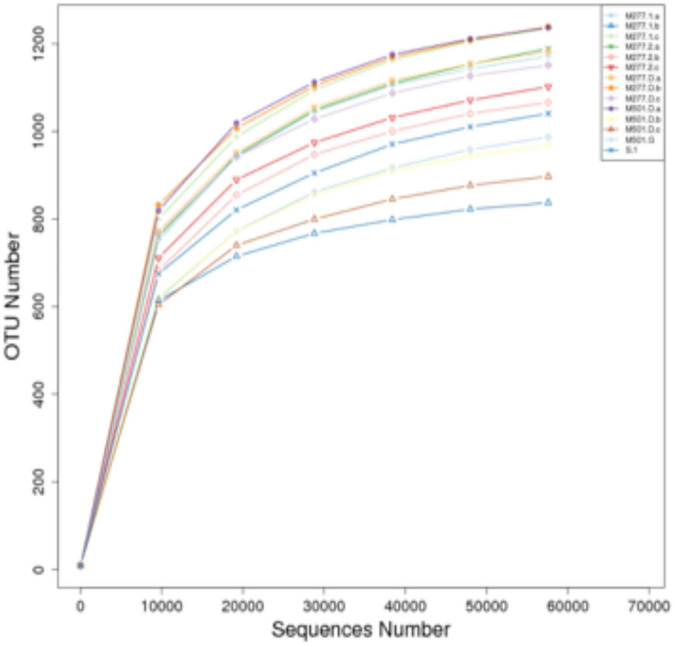
Dilution curve of soil samples.

**FIGURE 2:**
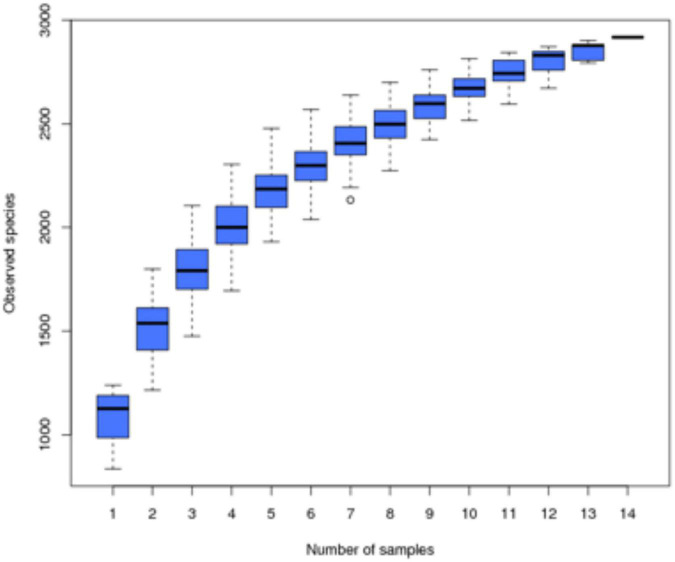
Species accumulation curve of soil samples.

Samples were subjected to high-throughput sequencing to obtain a total of 1,304,331 raw sequences, and after filtering, 1,220,837 high-quality effective sequences with an average length of 410–422 BP were selected. The two curves for determining data quality are the dilution curve and the species accumulation curve, which can measure whether the number of samples and the sequencing depth can effectively reflect the community structure in the samples (Rocchini et al., 2009; Li, 2011). The dilution curve of the sample (Figure 1) shows that with the increase of the amount of sequencing data, although there will still be new OTUs, the dilution curve of the sample has tended to be flat, indicating that the sequencing results basically cover the biological information of the vast majority of bacteria in the sample, which can more truly reflect the bacterial community diversity in the soil, and more sequences will only increase the limited species richness, it can be used for subsequent analysis. At the same time, the species accumulation curve of the sample (Figure 2) shows that as the sample size increases, the species accumulation curve of the sample also tends to be flat, and the species increase gradually decreases, indicating that the sequencing of 14 sample sizes is reliable and can be used for follow-up analysis.

### Analysis of the Composition of the Sample Microbiome

#### Community Composition Analysis of Samples at Phylum Level

**FIGURE 3:**
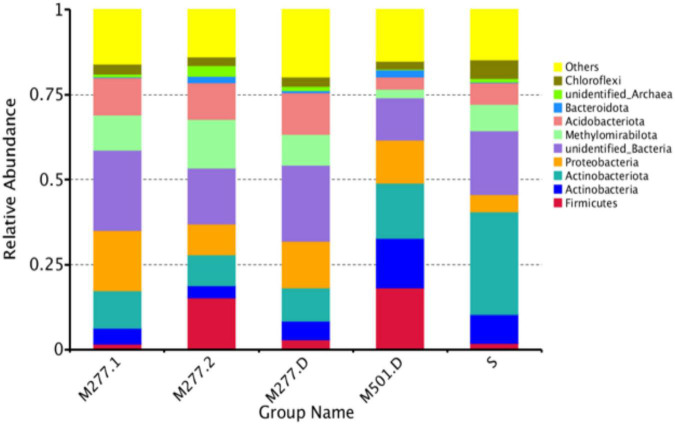
Species abundance histogram at the phylum level.

As shown in Figure 3, based on the phylum level analysis, there are 8 dominant phylum (relative abundance > 1%) in the tested samples, accounting for 64.10% of all microorganisms, and 20.06% of undetected microorganisms. There were 7 dominant phyla (relative abundance > 1%) in M277 soil samples, which were Proteobacteria (13.49%), Acidobacteriota (11.31%), Methylomirabilota (11.24%), Actinobacteriota (9.85%), Firmicutes (6.65%), Actinobacteria (4.61%), and Chloroflexi (2.79%). The dominant phyla of M501 soil samples were Firmicutes (18.24%), Actinobacteriota (16.09%), Actinobacteria (14.57%), Proteobacteria (12.76%), Acidobacteriota (3.52%), Methylomirabilota (2.58%), Chloroflexi (2.31%) and Bacteroidota (2.24%). There were 7 dominant bacteria (relative abundance > 1%) in the control soil samples, which were Actinobacteriota (30.14%), Firmicutes (18.62%), Actinobacteria (8.49%), Methylomirabilota (7.76%), Acidobacteriota (6.51%), Chloroflexi (5.42%), and Proteobacteria (5.15%).

#### Community Composition Analysis of Samples at Genus Level

**FIGURE 4:**
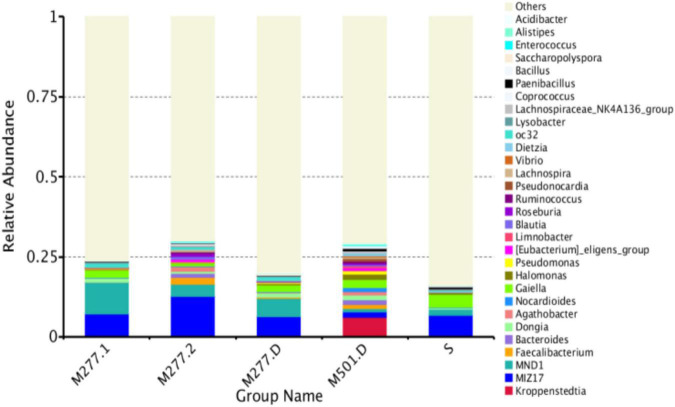
Species abundance histogram at genus level (TOP30).

As shown in Figure 4, there are fewer identifiable bacterial genus, and the samples tested had a total of 13 dominant bacterial genus (relative abundance > 1%), accounting for 20.22% of all microorganisms. There were 4 dominant species (relative abundance > 1%) in M277 soil samples, which were MIZ17 (8.66%), MND1 (6.47%), Gaiella (1.87%) and oc32 (1.00%). The dominant genera of M501 soil samples were Kroppenstedtia (6.23%), Gaiella (2.38%), Halomonas (1.89%), MIZ17 (1.65%), Bacteroides (1.60%), Dongia (1.51%), Faecalibacterium (1.32%), Nocardioides (1.29%), Pseudomonas (1.04%) and Pseudonocardia (1.00%). There were three dominant genera (relative abundance > 1%) in the control soil samples, which were MIZ17 (6.65%), Gaiella (4.00%) and MND1 (2.08%).

### Analysis of the α Diversity of Microbiomes

The closer Good’s Coverage is to 1, the closer the sequencing depth is to the true value. According to the data in Table 2, the sequencing results of each sample are true and reliable.

**TABLE 2:** Alpha diversity index of soil samples.

Group	Name	Shannon index	Simpson index	Chao1 index	ACE	Good’s coverage/%	Observed-species
S	s.1	7.529	0.987	1107.306	1146.927	0.997	1041
M277.1	M277.1.a	6.922	0.980	1073.776	1087.482	0.997	987
	M277.1.b	7.512	0.988	879.632	878.779	0.999	837
	M277.1.c	7.697	0.987	1299.595	1317.929	0.997	1235
M277.2	M277.2.a	7.663	0.986	1261.462	1281.904	0.997	1189
	M277.2.b	6.880	0.968	1130.008	1132.319	0.998	1066
	M277.2.c	7.256	0.978	1170.542	1178.509	0.998	1102
M277.D	M277.D.a	7.527	0.986	1249.603	1254.627	0.998	1182
	M277.D.b	8.056	0.992	1313.007	1324.111	0.997	1239
	M277.D.c	7.712	0.988	1215.008	1216.070	0.998	1151
M501.D	M501.D.a	7.122	0.952	1300.609	1301.509	0.998	1237
	M501.D.b	7.363	0.985	1020.248	1033.930	0.998	969
	M501.D.c	7.257	0.982	956.272	958.499	0.998	897
	M501.G	6.759	0.947	1242.000	1242.559	0.998	1171

Bacterial community richness is expressed in the Chao1 and ACE indices, and the diversity of bacterial communities is expressed in the Shannon and Simpson indices. The diversity and richness index of soil bacterial community of m277 are shown in Table 2. The order of Chao1 and ACE index is M277.D > M277.2 > M277.1; the Shannon and Simpson index is in the order of M277.D > M277.1 > M277.2, but the overall data are not much different. Comparing the diversity and richness of bacterial communities in the soil at the bottom of M277 and M501, the results showed that M277.D > M501.D.

### Operational Taxonomic Units Clustering Analysis of Microbial Communities

**FIGURE 5:**
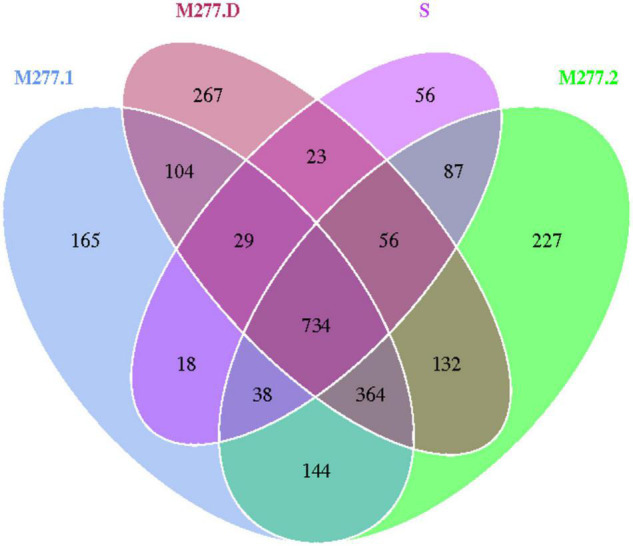
Wayne plot of OTU number in soil samples from M277.

**FIGURE 6:**
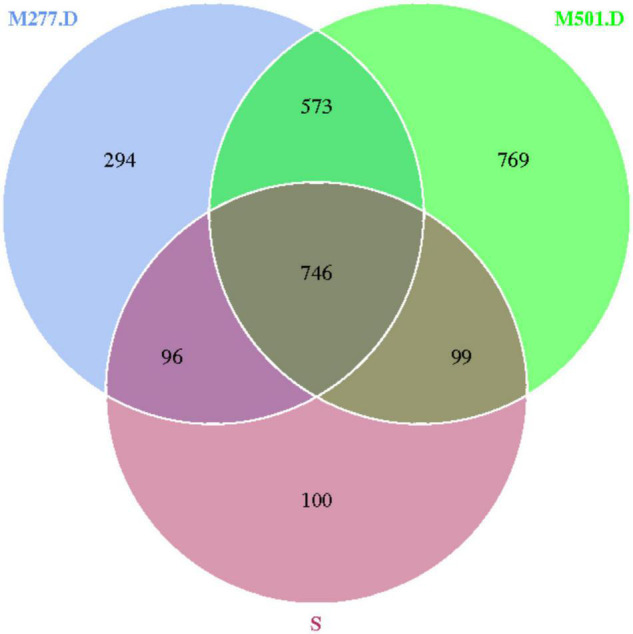
Wayne plot of microbial OTU in soil samples.

The 14 samples were totally divided into 5 groups, as shown in Figure 5, in which a total of 1,041 OTUs were obtained from Group S, 1,596 OTUs were obtained from group M277.1, 1,782 OTUs were obtained from group M277.2, 1,709 OTUs were obtained from group M277.D and 2,187 OTUs were obtained from group M501.D. The number of OTUs in M277 of different hierarchical grouped samples shared 2,388, but their shared OTUs shared 1,098. The number of OTUs contained in the sample set was regular as the number of OTUs contained in the sample S<M277.1<M277.2≈M277.D. As shown in Figure 6, there were 1,319 OTUs shared between M277. D and M501.D, 842 OTUs shared between M277.D and S, and 845 OTUs shared between S and M501.D. There were 294 unique OTUs in M277.D, 769 unique OTUs in M501.D, and 100 unique OTUs in S. The number of OTUs in these three groups was 2,677 in total, while the number of OTUs they collectively contained was 746. The number of OTUs contained in soil samples of the three groups followed the rule M501.D > M277.D > S.

### β Diversity Analysis

**FIGURE 7:**
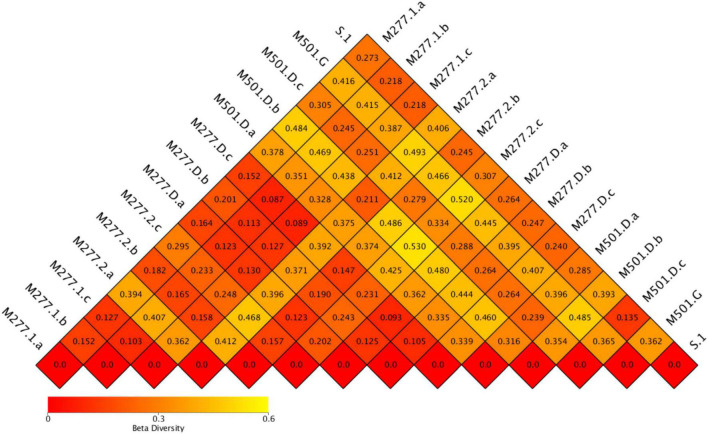
Beta diversity index heat map of soil samples in tombs.

The heat map of the sample (Figure 7) shows that the average values of the difference coefficients at five different sampling locations are 0.257 in group S, 0.228 in group M277.1, 0.302 in group M277.2, 0.223 in group M277.D and 0.345 in group M501.D. The results show that group M501.D is the largest and group M277.D is the smallest. It is speculated that M501 is a tomb that has been excavated for 1 year, which is quite different from M277 excavated on site, Therefore, the specificity of group M501.D was the strongest.

**FIGURE 8:**
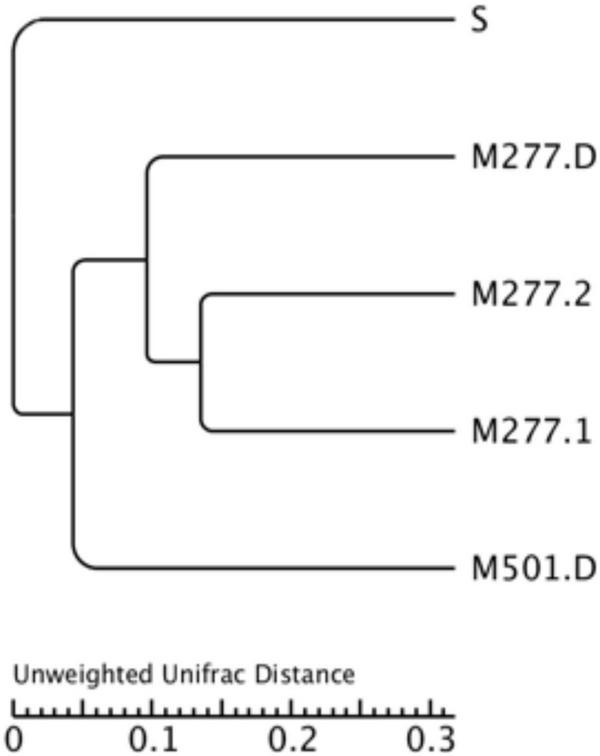
UPGMA clustering tree based on UniFrac.

Based on the analysis of sample similarity cluster tree in UniFrac, the samples under test are helpful to visualize the similarity and differences in the evolution of sample microorganisms in different tombs, and the evolutionary distance between samples can be directly observed by the distance of branches and the distance of clustering. As can be seen from Figure 8, three groups of M277 samples were first grouped into one category, the soil samples were the most similar, M277 and M501 were similar to the tomb, followed by the lowest similarity with the native samples.

**FIGURE 9:**
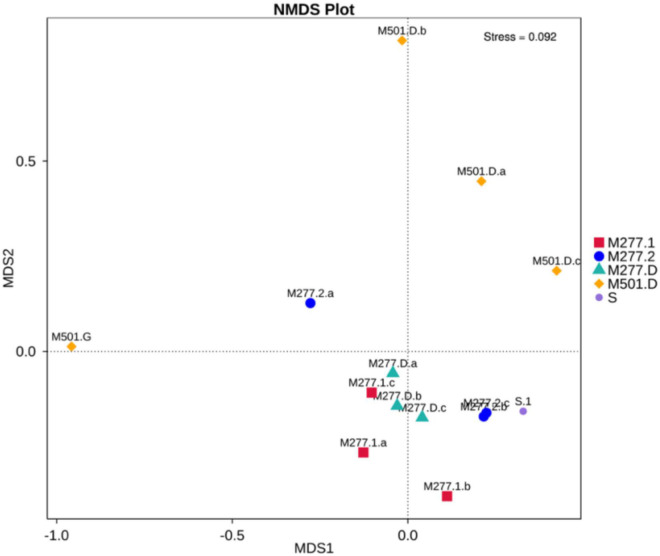
NMDS of microbial community structure and composition of samples.

Non-metric Multidimensional Scaling (NMDS) was used to analyze the differences of microbial communities between the soil samples of Yangguanzhai Cemetery (Figure 9). It can be seen that the microbial community structure between the soil samples of M277 and M501 tomb is significantly different. In group M501. D, because M501.G is a human bone sample, the microbial community structure is quite different from the other three soil samples, even in the same burial environment, the microbial community structure in different media is quite different, so the microbial structure of human bone itself needs to be further studied. The soil at different locations in M277 overlapped to some extent, and the three groups of samples had similar microbial community structure.

### LEfSe Analysis of Soil at the Bottom of Tombs Excavated One Year Apart

**FIGURE 10:**
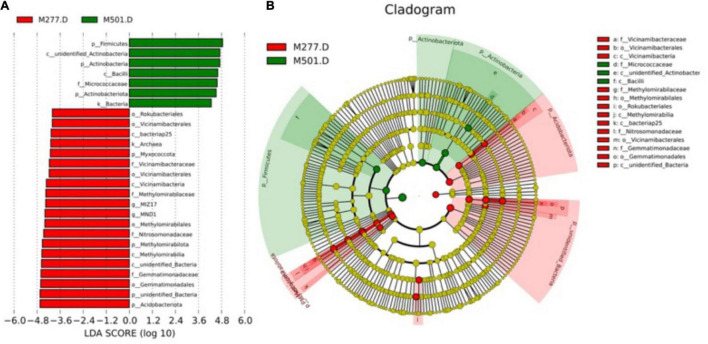
LEfSe analysis of soil samples. **(A)** Linear discriminant analysis (LDA) effect quantity distribution between different groups, the *X*-axis represents LDA score (log10), *Y*-axis indicates significantly different bacteria (LDA > 4.0). **(B)** The arc diagram of taxonomic evolutionary branches with significant differences between the two groups shows the subordination of different genera from the inside out. The node size corresponds to the average relative abundance of different genera. The red area is the distribution area of different genera in M277.D group and the green area is the distribution area of different genera in M501.D group.

Analysis of the differences in community structure between M277.D and M501.D by LEfSe (Figure 10) shows that there are 27 species with significant differences in soil at the bottom of the two tombs, of which M277.D has 20, M501.D has 7, and M277.D has significant differences in the number of species, but the abundance of the two is not much different. In M277.D, Myxococcota, Methylomirabilota and Acidobacteriota were the main soil microflora at phylum level, Gemmatimonadaceae, Methylomirabilaceae, Nitrosomonadaceae and Vicinamibacteraceae were the main soil microflora at family level, and MIZ17 and MND1 were the main soil microflora at genus level. In group M501.D, Firmicutes, Actinobacteria and Actinobacteriota were the main microflora at phylum level, and Micrococcaceae was the main microflora at family level.

## Discussion

Based on high-throughput Sequencing, the bacteria in the soil of Yangguanzhai Cemetery mainly belong to 8 dominant phyla, such as Proteobacteria, Actinobacteria and Firmicutes, and 13 dominant genera, such as Kroppenstedtia, Gaiella and MIZ17.

### Difference Analysis of Bacteria in Different Positions of M277

Operational taxonomic units cluster analysis of different depths of fill in m277 shows that the number of OTUs in the sample group is S < M277.1 < M277.2≈M277.D, the number of OTU in M277 grave filling is more abundant than that in raw soil, and with the increase of sampling depth, the number of OTU in soil samples is more abundant; The number of OTUs in M277.2 and M277.D at the same sampling depth is similar, and there are 1,286 OTUs in M277.2 and M277.D, which indicates that the total number of OTUs in tomb chamber filling and tomb passage filling at the same sampling depth is similar, and the similarity is high, with only a small number of unique OTUs.

The different depths of the same soil, due to the differences in water, nutrients, ventilation, temperature and other environmental factors and the characteristics of the microorganisms themselves, resulting in the vertical distribution of microorganism differences. Surface soil due to ultraviolet radiation and lack of water, microorganisms are prone to death and a small number, at depths of 5–20 cm, the largest number of microorganisms; From below 20 cm, the number of microorganisms decreased with the increase of depth (Le and Wang, 2020). It is different from the previous research results α Diversity analysis showed that the richness of bacterial community in the soil near human bone at the bottom of M277 was the highest, and that in the soil at 20 cm depth was the lowest, indicating that the richness of soil bacterial community increased with the increasing of sampling depth, and the closer the sampling point was to human bone, the higher the richness of soil bacterial community; The diversity of bacterial community in the soil near human bone at the bottom of M277 was the highest, and it was slightly lower at the depth of 20 and 30 cm, which was similar to the pattern of species richness. At the same time, there was no significant difference in the diversity and richness of the bacterial community among the sampling points, which indicated that the microbial distribution in the whole soil environment of Yangguanzhai Cemetery was relatively uniform.

### Changes of Soil Environment of Human Bones After Excavation

In order to monitor the change of soil microorganisms after archaeological excavation, the structure of soil microbiome near human bones at the bottom of M501 was compared, and the OTU clustering showed that M277.D and M501.D shared the majority of OTUs, only a small part of which was different, the consistency and difference between the two soil microbiomes was high, the difference was small, and the diversity and richness of the bacterial communities in the M277 soil were slightly greater than that of M501 bottom soil.

According to the LEfSe analysis of M277.D and M501.D, the species with significant difference between the two groups can be selected. It can be seen that after archaeological excavation, the soil near the human bone at the bottom of M501, which was exposed to the atmosphere for 1 year and experienced environmental mutation, not only reduced the diversity and complexity of bacterial community, but also produced new species with significant differences and disappeared the species with significant differences in the closed space soil at the bottom of M277, it is possible that the sudden exposure of soil to air after archaeological excavation will lead to the death of some bacteria, and the change of environment and the exchange with atmospheric microorganisms will also lead to the production of new bacteria.

### The Influence of Grave Microorganism on Human Bone

In the past, Tang Xianchun (Tang et al., 2003) used to isolate and identify microorganisms in the coffin of Marquis Yi Zeng’s tomb by traditional culture methods. The culturable bacteria include bacillus, Microbacterium and Flavobacterium, of which Bacillus accounts for the vast majority. Wu Fasi (Wu et al., 2011; Ma et al., 2018) used molecular biology technology to detect 6 genera of bacteria in the tombs of Wei and Jin Dynasties in Jiayuguan. Among them, Pseudonocardia and Acidobacteria are the dominant species, and fungi are three genera, namely Aspergillus, Phialosimplex and lateral tooth fungus. Zhang Hui (Zhang et al., 1998) isolated and identified eight genera of fungi from Han tombs, and Penicillium was the dominant group. Wu Fasi (Wu et al., 2012) analyzed the community composition of the corrosion fungi on the surface of the archaeological excavation site of the Western Zhou Dynasty cemetery in Dahekou, and found that the main fungi causing microbial corrosion were Pseudobursa, Alternaria and Malus; He also analyzed the fungal community composition of Xu Xianxiu’s tomb in Taiyuan, and found that Pleurotus albus and Penicillium villosum were the dominant species (Wu et al., 2016). It can be seen that these studies mainly focus on the identification of microbial species in the burial environment.

This paper attempts to reveal the influence of microorganisms in buried environment on human bone preservation. According to the archaeological excavation, most of the human bones in the early tombs of Yangguanzhai Cemetery are buried directly in the soil without burial tools. Usually, soil has good ecological conditions suitable for microbial survival, so the growth, reproduction and metabolism of microorganisms in soil are relatively vigorous. In the process of survival, human bones in tombs provide the necessary carbon and nitrogen sources for microorganisms. In addition, the secreted products and metabolites of microorganisms in the soil will also decompose human bone, and the secreted pigments will affect the color of human bone itself, which will have an impact on the preservation and research of human bone in the future.

In Yangguanzhai Cemetery, 94 soil samples were tested for pH, and the pH value was between 8.40 and 9.33, with an average value of 8.95, which was alkaline soil. From the tombs of Yangguanzhai Cemetery, a large number and complex species of dominant bacteria were detected, especially Proteobacteria and Firmicutes, which were the most widespread species in the burial soil of Yangguanzhai Cemetery. Proteobacteria is the largest group in the whole bacterial domain with complex species and wide distribution. Most Proteobacteria are facultative or obligate anaerobes and heterotrophic or autotrophic chemical energy organisms, rich species diversity and genetic diversity make Proteobacteria have a wide range of physiological and metabolic pathways (Ciccarelli et al., 2006; Song et al., 2016). Firmicutes is mainly composed of Bacillus and Clostridium. Many Firmicutes can form spores and are in the dormant stage of inactivity, severe dehydration and high resistance to environmental pressure. They have a high survival rate, and Firmicutes metabolic activities produce acetate and lactate, which have a continuous impact on bones. Bacillus has strong environmental adaptability, and Clostridium has strong degradation ability and metabolic activity (Brown et al., 1984; Song et al., 2015). Bacteroidetes with the same high content has outer membrane, peptidoglycan layer and plasma membrane. Bacteroidetes is one of the most oxygen resistant anaerobic bacteria, which carries out anaerobic respiration. The main by-products are acetic acid, isovaleric acid and succinic acid. Then actinomycetes appear as spores or nutrients in different habitats such as soil, aquatic environment, plant litter, compost and food. It plays an important role in the decomposition of organic matter, thus plays an important role in the turnover of organic matter and carbon cycle. It is an important part of humus formation and has a great impact on the corrosion of bones. Moreover, there are more kinds of microorganisms near human bone, which indicates that human bone is obviously affected by microorganisms.

In addition, in the process of human bone burial, because the environment is relatively closed and gradually tends to be stable, the species of microorganisms do not change greatly after they act on human bone. However, due to the sudden change of environment, the diversity of microorganisms in the buried soil has changed to some extent. Therefore, in the follow-up process of human bone preservation, we need to take some protective measures to prevent the impact of microorganisms on human bone.

## Conclusion

For the first time, high-throughput sequencing method was used to determine the microbial community structure and diversity in the burial soil of Yangguanzhai Cemetery. Eight dominant phyla were found, but there are still many new species to be further identified.

The diversity analysis of samples microorganisms showed that the overall distribution of soil microorganisms in Yangguanzhai Cemetery is relatively uniform, and after the archaeological excavation of the tomb, the human bone burial environment was mutated, and the microbiome in the overall buried environment before and after the mutation was more similar, but there were still more new species with significant differences.

The highest richness and diversity of microbial community structure were found in the soil near human bones, which indicated that microorganisms played an important role in the corrosion process of human bones. Therefore, some measures should be taken to prevent and control microorganisms and protect human bones.

## Data Availability Statement

The datasets presented in this study can be found in online repositories. The names of the repository/repositories and accession number(s) can be found below: The 16S rRNA gene sequence data from the present study have been archived at the NCBI Sequence Read Archive (SRA) under the BioProject accession number PRJNA790823.

## Author Contributions

XL, XZ, and MS designed the research study. XW, LY, MC, and ZH conducted the research. XW, XL, and JZ analyzed the data. XW and XL prepared all the figures and wrote the main manuscript. All authors contributed to the article and approved the submitted version.

## Conflict of Interest

The authors declare that the research was conducted in the absence of any commercial or financial relationships that could be construed as a potential conflict of interest.

## Publisher’s Note

All claims expressed in this article are solely those of the authors and do not necessarily represent those of their affiliated organizations, or those of the publisher, the editors and the reviewers. Any product that may be evaluated in this article, or claim that may be made by its manufacturer, is not guaranteed or endorsed by the publisher.
